# *Helicobacter pylori* filtrate impairs spatial learning and memory in rats and increases β-amyloid by enhancing expression of presenilin-2

**DOI:** 10.3389/fnagi.2014.00066

**Published:** 2014-04-11

**Authors:** Xiu-Lian Wang, Ji Zeng, Jin Feng, Yi-Tao Tian, Yu-Jian Liu, Mei Qiu, Xiong Yan, Yang Yang, Yan Xiong, Zhi-Hua Zhang, Qun Wang, Jian-Zhi Wang, Rong Liu

**Affiliations:** ^1^Key Laboratory of Neurological Disease, Ministry of Education, Department of Pathophysiology, Tongji Medical College, Huazhong University of Science and TechnologyWuhan, China; ^2^Department of Pathology, Hubei University of Chinese MedicineWuhan, China; ^3^Department of Clinical Laboratory, Wuhan Pu Ai Hospital, Huazhong University of Science and TechnologyWuhan, China

**Keywords:** Alzheimer's disease, *Helicobacter pylori*, learning, memory, Aβ_42_, presenilin-2

## Abstract

*Helicobacter pylori (H. pylori)* infection is related with a high risk of Alzheimer's disease (AD), but the intrinsic link between *H. pylori* infection and AD development is still missing. In the present study, we explored the effect of *H. pylori* infection on cognitive function and β-amyloid production in rats. We found that intraperitoneal injection of *H. pylori* filtrate induced spatial learning and memory deficit in rats with a simultaneous retarded dendritic spine maturation in hippocampus. Injection of *H. pylori* filtrate significantly increased Aβ_42_ both in the hippocampus and cortex, together with an increased level of presenilin-2 (PS-2), one key component of γ-secretase involved in Aβ production. Incubation of *H. pylori* filtrate with N2a cells which over-express amyloid precursor protein (APP) also resulted in increased PS-2 expression and Aβ_42_ overproduction. Injection of *Escherichia coli* (*E.coli*) filtrate, another common intestinal bacterium, had no effect on cognitive function in rats and Aβ production in rats and cells. These data suggest a specific effect of *H. pylori* on cognition and Aβ production. We conclude that soluble surface fractions of *H. pylori* may promote Aβ_42_ formation by enhancing the activity of γ-secretase, thus induce cognitive impairment through interrupting the synaptic function.

## Introduction

Alzheimer's disease (AD) is the most common type of dementia; patients show hippocampus-dependent spatial memory impairment in the incipient stage of the disease (Lithfous et al., 2013). Pathologically, AD is characterized by the deposition of extracellular senile plaques (SP) and formation of intracellular neurofibrillary tangles (NFT) within the afflicted brains (Braak and Braak, 1992). The SP are mainly composed of β-amyloid (Aβ), surrounded by dystrophic neuritis. Numerous studies suggest the Aβ toxicity in promoting the development of AD, such as influencing calcium homeostasis (Mattson et al., 1993; Wu et al., 1997), activating caspases (Harada and Sugimoto, 1999), stimulating protein phosphorylation (Busciglio et al., 1995), and causing mitochondrial abnormalities (Rui et al., 2006; Wang et al., 2008). Aβ is also reported to disrupt hippocampal synaptic plasticity (Walsh et al., 2002; Wang et al., 2002; Li et al., 2009), the latter, is supposed to be the base of hippocampus-dependent learning and memory (Muller et al., 2002). Thus, Aβ plays an important role in inducing cognitive impairment and AD-like pathologic changes. But till now the upstream factors that promoting Aβ overproduction in AD has not been fully elucidated.

Aβ is produced by the cleavage of amyloid precursor protein (APP) through β and γ-secretase. Abnormal enhanced activity of β and γ-secretase may underlie Aβ overproduction. It is well known that gene mutations of presenilin (PS)-1 and PS-2, key protein members of γ-secretase, are causative for increased Aβ production in familial AD (Borchelt et al., 1996; Duff et al., 1996; Citron et al., 1997; Xia et al., 1997). However, the mechanism leading to abnormal γ-secretase activation in the majority sporadic AD patients is still unclear.

*Helicobacter pylori* (*H. pylori*) is a gram-negative bacterium which chronically infects more than one half of the world's population. Recently, several clinical surveys and investigations suggest a possible relationship of *H. pylori* infection and AD development. AD patients have a higher prevalence of *H. pylori* than controls (Kountouras et al., 2006); increased levels of *H. pylori* antibodies are detected both in plasma and cerebrospinal fluid of AD patients (Malaguarnera et al., 2004; Kountouras et al., 2009a). AD patients infected by *H. pylori* tend to be more cognitively impaired (Roubaud-Baudron et al., 2012), and *H. pylori* eradication therapy has a beneficial effect on AD patients with *H. pylori* infection (Kountouras et al., 2009b, 2010). However, all these investigations are based on clinical observation, till now the direct laboratory evidence link *H. pylori* infection and AD is still lacking.

In the present study, we explored the effect of soluble *H. pylori* surface fractions on the cognitive function and Aβ production in rats. We found that intraperitoneal injection of *H. pylori* filtrate could induce spatial learning and memory impairment in rats, impair the maturation of spines, and increase Aβ_42_ production both in hippocampus and cortex, together with enhanced expression of PS-2. Thus, soluble surface fractions of *H. pylori* may promote Aβ_42_ production by enhancing the activity of γ-secretase, and induce cognitive impairment through interrupting the synaptic function.

## Materials and methods

### Antibodies and chemicals

Rabbit polyclonal antibodies (pAb) against N-methyl-D-aspartic acid receptor (NMDA)-NR2A, NR2B, NR1, postsynaptic density (PSD)-93, PSD-95, Pan-Cadherin (1:1000), and mouse monoclonal antibody (mAb) DM1A against α-tubulin (1:2000) were all from Abcam (Cambridge, UK). PAb against α-amino-3-hydroxy-5-methyl-4-isoxazolepropionic acid (AMPA)-receptor GluR1 (1:500) and mAb against AMPA-GluR2 (1:1000) were from Millipore (Billerica, MA, USA). PAbs against β-site APP cleaving enzyme (BACE)-1 and PS-2 (1:500) were from Santa Cruz (Santa Cruz, CA). MAb against PS-1 was from Chemicon (Temecula, CA). Secondary antibodies for Western blotting anti-rabbit or anti-mouse IgG conjugated to IRDyeTM were from Licor Biosciences (Lincoln, NE, USA). Other reagents were of the highest quality available and obtained from commercial sources.

### Preparation of *H. pylori* and *E. coli* filtrates

*H. pylori* strain TN2GF4 (Ohkusa et al., 2003) was a gift from Dr. Zhu Liang-ru (Department of Digestive Internal Medicine, Union Hospital, Huazhong University of Science and Technology), *E.coli* strain 25922 was from American Type Culture Collection (Manassas, VA, USA). *H. pylori* bacteria were plated onto Brucella agar supplemented with 5% horse blood (BBL, Becton Dickinson Microbiology, Cockeysville, MD, USA) and incubated at 37°C in a microaerophilic environment for 3–7 days. *E.coli* bacteria were plated onto blood agar (Columbia agar, bio-merieux, France) and incubated at 37°C for 24 h. The bacteria were harvested into pyrogen-free Dulbecco's PBS (Cellgro, Mediatech, Herndon, VA), then pelleted by centrifugation at 4000 g for 10 min, and bacterial numbers were determined by re-suspension in PBS to an OD600 nm of 1.5, corresponding to 3.6 × 10^8^ CFU/ml as described previously (Keates et al., 1999). Defined numbers of bacteria were then re-suspended in antibiotic free Opti-MEM/DMEM medium (1:1) medium for 30 min at 37°C, pelleted at 4000 g for 10 min, the supernatants were then filtered through a 0.2 μm pore size filter (Acrodisc, Gelman, Ann Arbor, MI) and collected. The filtrates were diluted in Opti-MEM/DMEM medium (1:2) (we have previously demonstrated that *H. pylori* filtrate in this concentration could induce Alzheimer-like tau hyperphosphorylation) and stored at −20°C for use.

### Animal treatments and behavior test in morris water maze

**Figure 1:**
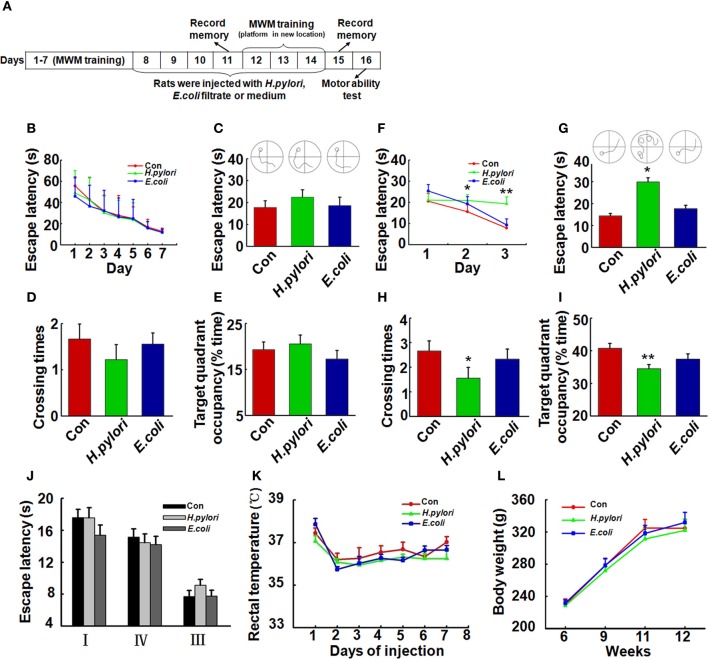
**Intraperitoneal injection *of H. pylori* filtrate induces spatial learning and memory impairment in rats. (A)** A schematic diagram for the treatment and behavior test of the rats. Thirty-four SD rats (male, 220 ± 20 g) were trained in Morris water maze (MWM) for 7 days, 27 rats which could find the hidden platform within 15 s at the end of training were selected and divided into three groups (*n* = 9 for each group) randomly. The rats were then intraperitoneally injected with *H. pylori, E.coli* filtrate or DMEM/Opti-MEM medium (280 μl/rat/day) for 7 days. On day 11 of behavior test (day 4 of injection), the spatial memory of the rats in MWM was measured. The rats were then trained in MWM for another 3 days with altered location of the hidden platform (re-learning) for testing the spatial learning ability. On day 15 (1 day after the end of injection) the new spatial memory was measured. On day 16 the motor ability of the rats was detected by recording the escape latency to a visible platform in the MWM. At the end of behavior test, the rats were anesthetized and decapitated, and the hippocampal extracts or brain slices were prepared for further studies. **(B)** All the rats were trained to find the hidden platform within 15 s before the injection. **(C–E)** Intraperitoneal injection of *H. pylori* filtrate for 3 days does not impair the formed spatial memory in MWM. The escape latency **(C)**, number of crossing the platform **(D)** and the residence time in the target quadrant **(E)** were recorded on day 4 of injection, no difference was observed among different groups. **(F–I)** Intraperitoneal injection of *H. pylori* filtrate impairs the learning ability and memory of the rats in MWM with changed location of the platform. A new learning process was started on day 5 of injection, with the hidden platform placed in a new quadrant. Rat injected with *H. pylori* filtrate showed significantly extended latency in the second learning **(F)** (*p* = 0.033, *F* = 2.439 on new-learning day 2, *p* = 0.001, *F* = 6.387 on new-learning day 3). On the testing day (1 day after the end of injection), the rats showed extended escape latency **(G)** (*p* = 0.016, *F* = 4.526), decreased number of crossings **(H)** (*p* = 0.034, *F* = 3.39) and residence time in the target quadrant **(I)** (*p* = 0.005, *F* = 4.858). The motor ability of the rats was tested in MWM with a visible platform in quadrant **II**, the latency of the rats from quadrant **I, III**, and **IV** to the platform was recorded. The results displayed no difference among groups **(J)**. The body temperature **(K)** and weight **(L)** of the rats also showed no differences among groups. ^*^p < 0.05, ^**^p < 0.01 vs. control group (mean ± SD, *n* = 9).

Three months old (220 ± 20 g) male Sprague Dawley rats (Grade: SPF) were supplied by the Experiment Animal Center of Tongji Medical College, Huazhong University of Science and Technology. All animal experiments were performed according to the “Policies on the Use of Animals and Humans in Neuroscience Research” revised and approved by the Society for Neuroscience in 1995. The proposal and experimental design were reviewed and approved by the Institutional Ethics Committee of Tongji Medical College, Huazhong University of Science and Technology. The rats were kept at 22 ± 2°C on daily 12 h light-dark cycles and received food and water *ad libitum*. The rats (*n* = 34) were pre-trained in Morris water maze (MWM) (Morris, 1984) to search a hidden platform under the water for 7 days. At the end of pre-training, rats which could find the platform within 15 s were selected and randomly divided into three experimental groups (*n* = 9 for each group) and received intraperitoneal injection of *H. pylori, E.coli* filtrate or the same volume of DMEM/Opti-MEM medium (1:1) as control (280 μl/rat/day) for 7 days. On day 4 of injection the spatial memory of the rats in the MWM was measured. Then the rats were trained again in MWM for 3 days, with the platform placed in a new quadrant (re-learning). Spatial memory retention for the second learning was measured 24 h later (day 8, one day after the last injection). On day 9, motor ability of the rats was tested in the MWM with a visible platform. The rats were then deeply anesthetized and decapitated, and the hippocampal extracts or brain slices were prepared for further studies. The timeline of the behavior test is described in Figure 1A.

### Nissl staining

The rats (*n* = 3) were deeply anesthetized with intraperitoneal injection of chloral hydrate (1 g/kg) and then fixed by transcardial perfusion with 0.9% NaCl, followed by 4% paraformaldehyde in 100 mM phosphate buffer (PB). After perfusion, the brains were postfixed in the same solution overnight at 4°C. Coronal sections of the brain were cut (30 μm thick) using Vibratome (Leica, S100, TPI), soaked in 1% toluidine blue for 3 min. Sections were then dehydrated using 95% and 100% ethanol solutions, transparented using xylene, placed under cover slips and analyzed with a microscope (Nikon, 90i, Tokyo, Japan).

### Golgi staining

The rats (*n* = 3) were deeply anesthetized and then fixed by transcardial perfusion with 0.5% NaNO_2_ followed by 4% formaldehyde and potassium dichromate with chloral hydrate which were mixed in 4% formaldehyde. After perfusion, the brains were postfixed in potassium dichromate with chloral hydrate mixed liquid for 3 days. Then the brains were moved into 1% AgNO_3_ solution for 3 days. Coronal sections of the brain were cut (30 μm thick) using Vibratome (Leica, S100, TPI). Sections were dehydrated using a graded series of ethanol solutions, transparented using xylene, placed under cover slips and analyzed with a microscope (Nikon, 90i, Tokyo, Japan).

### Cell culture and treatment

N2a/APP (N2a stably transfected with human APP) cells were grown to 70–80% confluence in 6-well culture plates in a DMEM/Opti-MEM medium (1:1) supplemented with 5% fetal bovine serum (Gibco, Grand Island, NY, USA) in the presence of 200 mg/L G418 (Gibco, Grand Island, NY, USA). To minimize stress responses induced by serum deprivation, cells were switched to 0.5% fetal bovine serum media for 1 day, kept in fresh serum-free media for 2 h. Then the cells were incubated with the prepared *H. pylori* filtrate, *E.coli* filtrate (2 ml/well), or DMEM/Opti-MEM medium for 24 h. At the end of incubation, all media were collected and centrifuged at 2000 g for 20 min, the supernatants were stored at −80°C for enzyme linked immunosorbent assay (ELISA); cells were rinsed twice in ice-cold PBS (pH 7.5) and collected, half of the cells were lysed with phosphate buffered saline (pH 7.5) containing 0.5 mM PMSF and 1:1000 protease inhibitor cocktail (Sigma-Aldrich, St. Louis, MO, USA), repeatedly frozen and thawed for three times and centrifuged at 2000 g for 20 min, the supernatants were collected and stored at −80°C for ELISA; other half of the cells were lysed with buffer containing 2 mM EGTA, 0.5 mM PMSF, 5 mM EDTA, 150 mM NaCl, 50 mM Tris-HCl (pH 7.4), 1% Triton X-100, and protease inhibitor cocktail (1:200), followed by sonication for 15 times on ice. The samples were stored at −80°C for Western blotting.

### Brain tissue homogenate and membranous protein extraction

Rat hippocampus and cortex were isolated and homogenized in 10 volumes (ml/g wet tissue) homogenate buffer containing 50 mM Tris-HCl, pH 7.0, 0.5 mM PMSF, 2.5 mM EDTA, 2.5 mM EGTA, 2.0 mM Na_3_VO_4_,100 mM NaF and 1:1000 protease inhibitor cocktail (Sigma-Aldrich, St. Louis, MO, USA). Then the homogenates were sonicated and stored at −80°C for Western blotting. The membrane proteins were extracted by using the membrane protein extraction kit P0033 from Beyotime (Shanghai, China) according to the manufacturer's instruction.

### ELISA

Sandwich ELISA was performed to measure the levels of Aβ_42_ and Aβ_40_ both in rat brain extracts, N2a/APP cell lysates and media by using the human Aβ_42_ ELISA kit E-EL-H0542 and human Aβ_40_ ELISA kit E-EL-H0543 (Elab, Wuhan, China) according to the manufacturer's instruction. Microplates were scanned with a microplate reader (Biotek, Winooski, VT, USA) set to 450 nm.

### Western blotting

The protein concentrations of the brain extracts and cell lysates were determined by BCA Protein Assay Kit (Thermo Fisher Scientific, Rockford, IL, USA). Then the samples were mixed with sample buffer containing 50 mM Tris-HCl (pH 7.6), 2% SDS, 10% glycerol, 10 mM dithiothreitol, and 0.2% bromophenol blue and boiled for 5 min. Boiled protein samples (15–20 μg per lane) were loaded and separated by 10% sodium dodecyl sulfate-polyacrylamide gel electrophoresis (SDS-PAGE), and then transferred to nitrocellulose membranes. The membranes were detected by using anti-rabbit or anti-mouse IgG conjugated to IRDye (800CW; Li-cor Biosciences, Lincoln, NE, USA) for 1 h at room temperature and visualized using the Odyssey Infrared Imaging System (Li-cor Biosciences, Lincoln, NE, USA). The protein bands were quantitatively analyzed by Kodak Digital Science 1D software (Eastman Kodak Company, New Haven, CT, USA).

### Statistical analysis

Data are expressed as mean ± SD and analyzed using SPSS 16.0 statistical software (SPSS Inc., Chicago, IL, USA). The One-Way analysis of variance (ANOVA) procedure followed by LSD's *post-hoc* tests was used to determine the differences among groups, *p* < 0.05 was considered as significant, *p* < 0.01 was considered as very significant.

## Results

### *H. pylori* filtrate induces spatial learning and memory impairment in rats

To evaluate the effect of *H. pylori* infection on learning and memory *in vivo*, we first trained the 3-month-old SD rats (*n* = 34) in the water maze for 7 consecutive days, then selected the rats (*n* = 27) which learned to find the hidden platform within 15 s for the following bacterial filtrates injection and detection (Figure 1A). As it was shown in Figure 1B, 27 rats which were able to find the hidden platform within 15 s were randomly divided into three groups (*n* = 9 for each group), each group showed the similar spatial learning and memory before the bacterial filtrates injection. Intraperitoneal injection of *H. pylori* filtrate for 3 days did not influence the formed spatial memory in the MWM before the injection (Figures 1C–E). But in a following new spatial learning task, compared with the controls, rats injected with *H. pylori* filtrate showed significantly prolonged latency in searching the hidden platform in a new quadrant, indicating an impaired spatial learning ability in the rats (Figure 1F, *p* = 0.033, *F* = 2.439 on new-learning day 2, *p* = 0.001, *F* = 6.387 on new-learning day 3). At the end of injection, the spatial memory for the new learning was test, rats injected with *H. pylori* filtrate for 7 days showed increased escape latency (Figure 1G, *p* = 0.016, *F* = 4.526), reduced crossing times and target quadrant occupancy compared with control and *E.coli* filtrate-injected rats (Figure 1H, *p* = 0.034, *F* = 3.39; Figure 1I, *p* = 0.005, *F* = 4.858). These data identified that *H. pylori* filtrate impairs spatial learning and memory. When the rats were trained to find a visible platform, they showed indistinguishable latency in the MWM (Figure 1J), indicating that the spatial learning and memory deficit in the *H. pylori* filtrate-injected rats is not caused by altered motivation or ability to learn explicit information. The body temperature and weight of the animals showed no difference among the groups (Figures 1K,L). In a summary, these behavior testing results suggest that intraperitoneal injection *H. pylori* filtrate induces spatial learning and memory deficit in rats.

### Intraperitoneal injection of *H. pylori* filtrate causes Aβ_42_ elevation in rat brains

**Figure 2:**
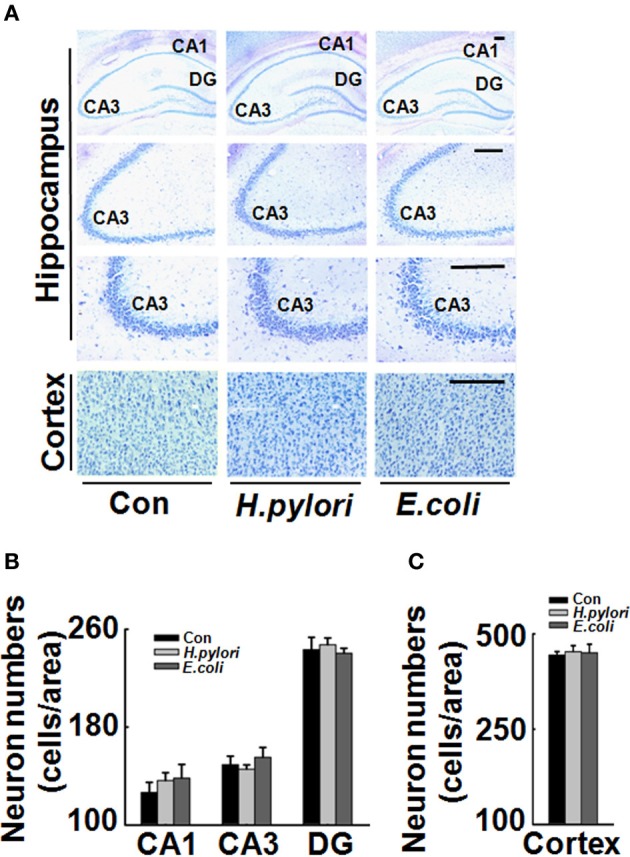
**Intraperitoneal injection *H. pylori* filtrate does not cause neuron death in rat brains**. SD rats were intraperitoneally injected with *H. pylori* filtrate, *E.coli* filtrate or DMEM/Opti-MEM medium (280 μl/rat/day) for 7 days as described in Figure 1A. Then the rats were anesthetized and fixed by transcardial perfusion (*n* = 3). Neurons in the rat brain were stained by Nissl staining. **(A)** Representative images from the hippocampus and cortex (Scale bars = 100 μm). Quantitative analysis of the neuron numbers in CA1, CA3, and DG region of hippocampus **(B)** and in the cortex **(C)** showed no difference among different groups.

**Figure 3:**
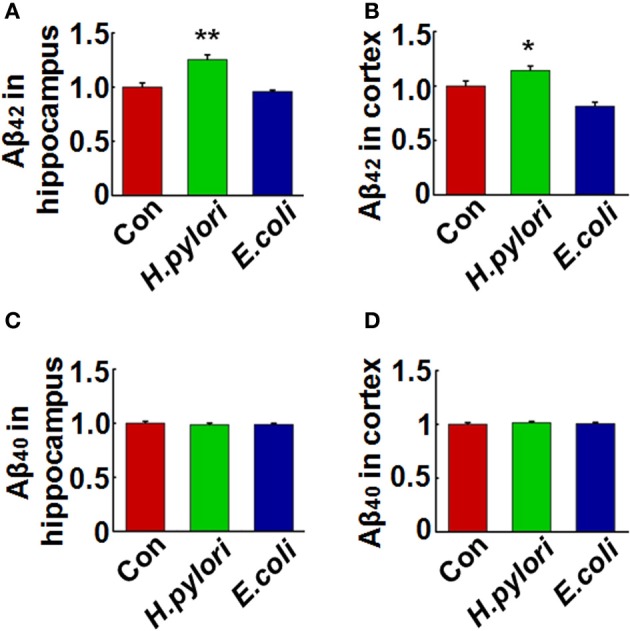
**Intraperitoneal injection of *H. pylori* filtrate causes Aβ_42_ elevation in rat brains**. SD rats were intraperitoneally injected with *H. pylori* filtrate, *E.coli* filtrate or DMEM/Opti-MEM medium (280 μl/rat/day) for 7 days as described in Figure 1A. The rats were then anesthetized and decapitated and the levels of Aβ_42_ and Aβ_40_ in the hippocampus and cortex were measured by ELISA **(A–D)**. Aβ_42_ levels both in the hippocampus (*p* = 0.002, *F* = 20.142) and cortex (*p* = 0.045, *F* = 16.637) were significantly increased in rats injected with *H. pylori* filtrate. ^*^p < 0.05, ^**^p < 0.01 vs. control group (mean ± SD, *n* = 9).

To explore the mechanisms underlying the spatial learning and memory deficit, we first detected whether there was a neuronal loss in the rat brains. Nissl staining of the neurons showed comparable cell number and density in the hippocampus and cortex of the rat brains in all the three groups (Figure 2), indicating that the learning and memory impairment in *H. pylori* filtrate-injected rats is induced by disturbed neuronal function but not by neuron loss. Aβ level is increased in AD brains and induces cognitive deficits in AD animal models (Billings et al., 2005; Liu et al., 2013). To further disclose the underlying mechanisms for memory deficit induced by *H. pylori* filtrate, we detected the Aβ_40_ and Aβ_42_ levels in the rat brains. The results showed that *H. pylori* filtrate injection induced Aβ_42_ elevation both in the hippocampus (Figure 3A, *p* = 0.002, *F* = 20.142) and cortex (Figure 3B, *p* = 0.045, *F* = 16.637), with no effect on Aβ_40_ levels (Figures 3C,D). Compared with Aβ_40_, Aβ_42_ is more toxic and specifically induces memory impairment in water maze and passive avoidance tests in mice (Jhoo et al., 2004), thus, *H. pylori* filtrate may cause learning and memory deficit through enhancing Aβ_42_ production in rat brains.

### Intraperitoneal injection of *H. pylori* filtrate impairs the dendritic spine maturation and reduces membrane expression of synaptic proteins in rat hippocampus

**Figure 4:**
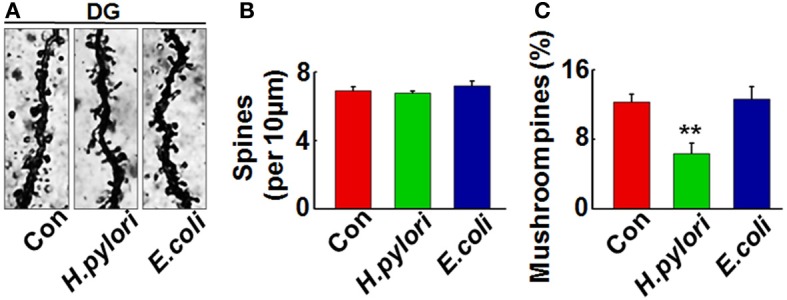
**Intraperitoneal injection of *H. pylori* filtrate impairs the dendritic spine maturation in rat hippocampal dentate gyrus**. SD rats were intraperitoneally injected with *H. pylori* filtrate, *E.coli* filtrate or DMEM/Opti-MEM medium (280 μl/rat/day) for 7 days as described in Figure 1A. Three rats were then anesthetized and fixed by transcardial perfusion, and brain slices of the rats were stained by Golgi staining. **(A)** The representative images of dendritic spines in the hippocampal dentate gyrus. Quantitative analysis of the spine density (calculated as the average number of spines per 10 μm on the dendrites) showed no difference among the groups **(B)**, while the percentage of mushroom spines were significantly decreased in rats injected with *H. pylori* filtrate **(C)** (*p* = 0.0003, *F* = 45.103). ^**^p < 0.01 vs. control group (mean ± SD, *n* = 3).

**Figure 5:**
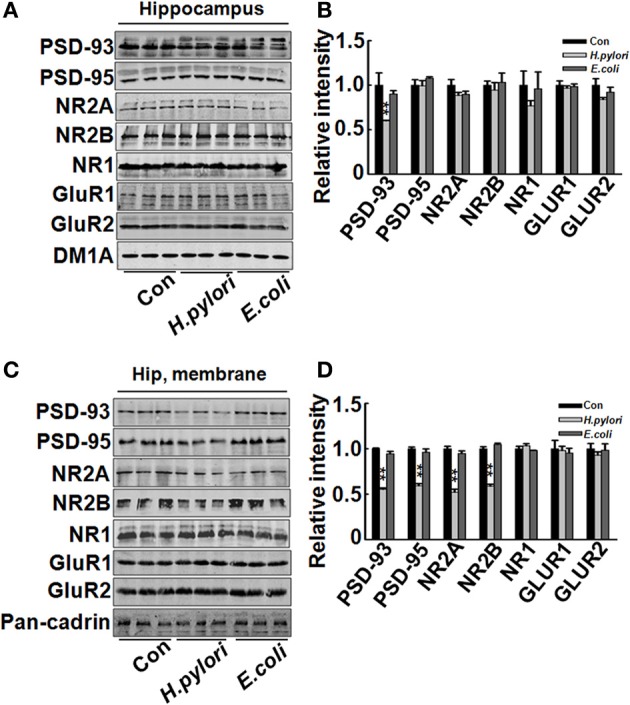
**Intraperitoneal injection of *H. pylori* filtrate induces decreased expression of synaptic proteins in rat hippocampus**. SD rats were intraperitoneally injected with *H. pylori* filtrate, *E.coli* filtrate, or DMEM/Opti-MEM medium (280 μl/rat/day) for 7 days as described in Figure 1A. Three rats were then anesthetized and decapitated and extracts of rat hippocampus were prepared and the synaptic receptor and scaffolding protein levels were measured by Western blotting with site-specific antibodies. DM1A and Pan-Cadherin were loaded as cytoplasm and membrane protein controls separately. Fifteen micrograms of protein samples were loaded for PSD-93, PSD-95, NR2A, NR2B, and Pan-Cadherin, 20 μg of proteins were loaded for NR1, GluR1 and GluR2, and 5 μg for DM1A. **(A,B)** The total protein level of PSD-93 was significantly decreased in *H. pylori* filtrate-injected rat brains (*p* = 0.004, *F* = 10.015), while PSD-95, NR2A, NR2B, NR1, GluR1, and GluR2 showed no difference among the three groups (**A**, representative bands; **B**, quantitative analysis of the density). **(C,D)** The membrane proteins in the rat hippocampus were extracted and synaptic proteins were detected by Western blotting. *H. pylori* filtrate injection induced remarkable decrease of membrane NR2A, NR2B, PSD-93, and PSD-95 levels (**C**, representative bands; **D**, quantitative analysis of the density, *p*-value: PSD-93, *p* = 0.002, *F* = 13.189; PSD-95, *p* = 0.001, *F* = 18.183; NR2A, *p* = 0.001, *F* = 21.32; NR2B, *p* = 0.008, *F* = 13.808). All the experiments were repeated at least three times. ^*^p < 0.01 vs. control group (mean ± SD, *n* = 3).

Impairment of synaptic plasticity contributes to learning and memory deficit. Aβ peptides may disrupt hippocampal synaptic plasticity via altered NMDA or AMPA receptor-PSD-MAGUK interactions (Proctor et al., 2011). Given this, we predict that *H. pylori* filtrate-induced Aβ_42_ elevation may cause learning and memory deficit through disturbing synaptic plasticity. To confirm this hypothesis, we observed the density and morphology of dendritic spines in the dentate gyrus of the hippocampus, one critical brain region involved in spatial learning and memory (Kesner, 2013). *H. pylori* filtrate-injected rats showed no difference of the total spine numbers compared with the other two groups, but the mature mushroom spines were significantly reduced (Figure 4, *p* = 0.0003, *F* = 45.103). Correspondingly, the membrane expression of functional synaptic receptors and scaffolding proteins such as NMDA-NR2A, NR2B, PSD-93, and PSD-95 was remarkably decreased in the hippocampus (Figures 5C,D, NR2A, *p* = 0.001, *F* = 21.32; NR2B, *p* = 0.008, *F* = 13.808; PSD-93, *p* = 0.002, *F* = 13.189; PSD-95, *p* = 0.001, *F* = 18.183), the total protein level of PSD-93 was also decreased in the *H. pylori* filtrate treated group (Figures 5A,B, *p* = 0.004, *F* = 10.015). These results suggest that *H. pylori* filtrate may induce cognitive deficit through disrupting synaptic plasticity.

### *H. pylori* filtrate increases Aβ_42_ production by enhancing the expression of γ-secretase

**Figure 6:**
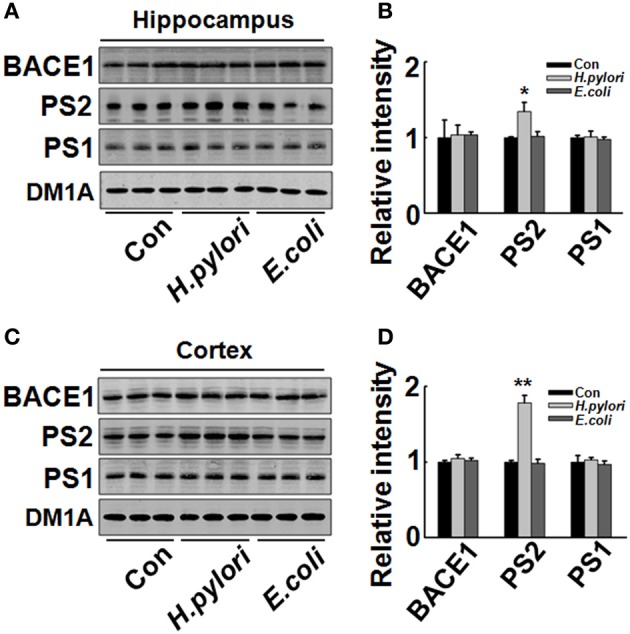
**Intraperitoneal injection of *H. pylori* filtrate induces increased expression of PS-2 in rat hippocampus**. The extracts of rat hippocampus and cortex were prepared and the protein levels of BACE-1, PS-1, and PS-2 were measured by Western blotting with site-specific antibodies. DM1A was used as protein loading control. Fifteen micrograms of the protein samples were loaded for BACE-1, PS-1, and PS-2 detection, and 5 μg for DM1A. **(A,C)** The protein levels of PS-2 were remarkably increased in *H. pylori* filtrate-injected rat hippocampus **(A)** and cortex **(C)**, while BACE-1 and PS-1 showed no difference among the three groups; **B** (*p* = 0.012, *F* = 8.24) and **D** (*p* = 0.004, *F* = 10.868) are the quantitative analysis. All the experiments were repeated at least three times. ^*^p < 0.05, ^**^p < 0.01 vs. control group (mean ± SD, *n* = 3).

**Figure 7:**
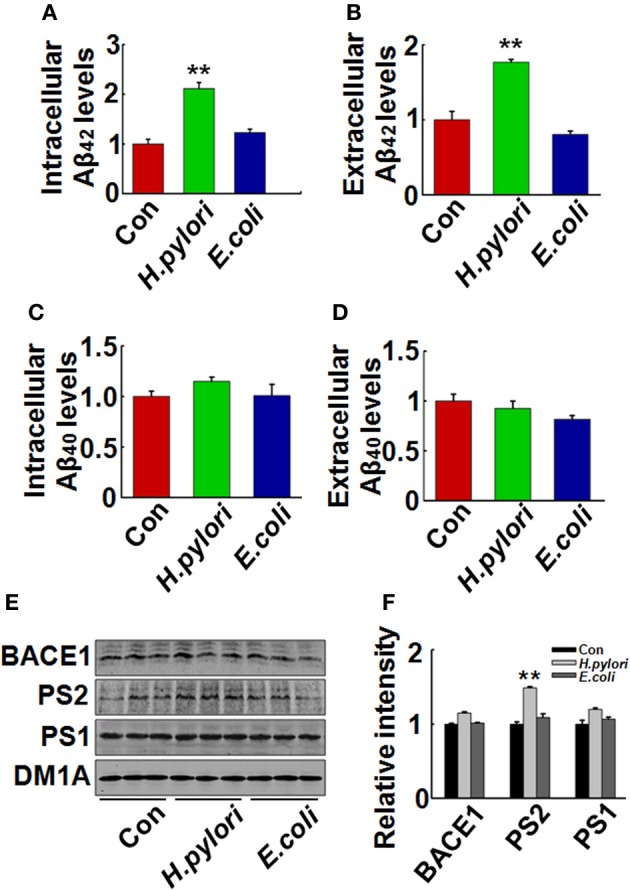
***H. pylori* filtrate increases Aβ_42_ production with enhanced expression of PS-2 in N2a/APP cells**. N2a/APPs cells were incubated with *H. pylori* or *E.coli* filtrate for 24 h. The levels of Aβ_42_ and Aβ_40_ in the medium and cell lysates were measured by ELISA **(A–D)**. *H. pylori* filtrate increased Aβ_42_ both in the cell lysates **(A)** (*p* = 0.001, *F* = 25.087) and the medium **(B)** (*p* = 0.002, *F* = 29.247) in N2a/APP cells, with no effect on Aβ_40_ levels **(C,D)**. The protein levels of PS-1, PS-2, and BACE-1 in the cell lysates were measured by Western blotting **(E,F)**. *H. pylori* filtrate incubation resulted in increased expression of PS-2 in cells. **E**, representative bands; **F**, quantitative analysis, *p* = 0.001, *F* = 22.92. All experiments were repeated at least three times. ^**^p < 0.01 vs. control group (mean ± SD, *n* = 9 for ELISA; *n* = 3 for Western blotting).

Aβ is released from the precursor protein APP through the cleavage of β and γ-secretase. To explore the mechanisms underlying the *H. pylori*-induced Aβ production, we test the expression of BACE-1, PS-1, and PS-2 in rat brains. We found that the protein level of PS-2, the key component of γ-secretase, was significantly increased in *H. pylori* filtrate-injected rat hippocampus (Figures 6A,B, *p* = 0.012, *F* = 8.24) and cortex (Figures 6C,D, *p* = 0.004, *F* = 10.868) while the BACE-1 and PS-1 levels remained unchanged (Figure 6). These data indicate that *H. pylori* filtrate may promote the Aβ_42_ production by enhancing the activity of γ-secretase. To further confirm this speculation, N2a cells stably over-expressing APP (N2a/APP) were incubated with *H. pylori* or *E.coli* filtrate for 24 h, then the Aβ levels, expression of β, and γ-secretase were detected. The results showed that *H. pylori* filtrate incubation directly increased intracellular Aβ_42_ level (Figure 7A, *p* = 0.001, *F* = 25.087) and promoted Aβ_42_ release into the culture media (Figure 7B, *p* = 0.002, *F* = 29.247), with a simultaneous up-regulation of PS-2 in cells (Figures 7E,F, *p* = 0.001, *F* = 22.92). No significant change of Aβ_40_ levels was observed among the different groups (Figures 7C,D). Thus, soluble exotoxins, or surface proteins released from the *H. pylori* bacteria may directly promote Aβ_42_ production and release by enhancing the activity of γ-secretase.

## Discussion

As the most common type of dementia, AD affects more than 35 million people in the world. The vast majority of AD cases are sporadic, implying that environmental factors are more causative in the disease development. *H. pylori*, a curved, spiral-shaped, gram-negative bacterium chronically colonizing in the stomach, has been linked to AD based on clinical surveys and investigations (Malaguarnera et al., 2004; Kountouras et al., 2007a), but the direct laboratory evidence is still lacking. In the present study, we demonstrated that *H. pylori* filtrate could induce AD-like cognitive deficit and Aβ_42_ overproduction possibly through enhancing the activity of γ-secretase.

*H. pylori* infection was first related to AD in a study performed by Malaguarnera et al. In this study, they reported a higher seropositivity for anti-*H. pylori* immunoglobulin G antibodies in 30 patients with AD than in 30 age-matched controls (Malaguarnera et al., 2004). In a later investigation by Kountouras et al., a higher prevalence of *H. pylori* infection in 50 AD patients than in 30 anemic controls was reported (Kountouras et al., 2006). Then they further observed increased *H. pylori* antibody in cerebrospinal fluid in AD (Kountouras et al., 2009a). In the following, two independent clinic studies indicated that *H. pylori* eradication regimen in AD patients was associated with decreased progression of dementia and a higher 5-year survival rate (Kountouras et al., 2010; Chang et al., 2013). On the other side, AD patients with *H. pylori* infection showed worse performance in cognition test and increased disease markers such as total/phosphorylated tau and cytokines in CSF compared with AD patients without *H. pylori* infection (Roubaud-Baudron et al., 2012; Beydoun et al., 2013). Thus, *H. pylori* may be one of the infectious etiologies of AD. However, till now the direct laboratory evidence that *H. pylori* are cause of AD is still lacking. One possible reason is that *H. pylori* infection may induce gastritis and peptic ulcer, which further cause hyperhomocysteinemia (Santarelli et al., 2004; Evrengul et al., 2007), the latter is related with a high risk of AD (Seshadri et al., 2002; Morris, 2003). *H. pylori* infection also results in the onset or progression of extradigestive disorders, such as polyradiculoneuropathy, hypertension, cardiovascular, and/or cerebrovascular ischemia, and stroke (Mendall et al., 1994; Blaser and Atherton, 2004; Kountouras et al., 2005; Sawayama et al., 2005). Most of these complications have been linked to AD. Thus, it is difficult to evaluate the direct effect of the bacteria *per se* on AD development. In the present study, through intraperitoneal injection of *H. pylori* filtrate, i.e., soluble surface fractions or other exotoxins secreted from the bacteria, we explored the effect of *H. pylori* on cognition and AD-like amyloidosis in rats.

AD patients first exhibit spatial learning and memory deficit in the progression of cognitive impairments. In our experiment, we found that intraperitoneal injection of *H. pylori* filtrate for 3 days did not interrupt the formed spatial memory in MWM. *H. pylori* is a bacterium chronically colonized to the stomach of the patient, the effect of *H. pylori* on the brain may also occur in a long time. We speculated a longer treatment may induce a difference. Thus, we prolonged the injection to 7 days, and trained the rats in MWM with changed location of the hidden platform. In the following new spatial learning, rats injected with *H. pylori* filtrate showed impaired performance compared with control rats. In a test of the newly-formed memory at the end of the training, these rats also exhibited worse memory ability compared with controls. To exclude the possibility that *H. pylori* filtrate may influence the performance of the rats in water maze through unspecific effects such as fever, decreased food intake, or impairment of the motor ability, we detected the body weight, temperature, and escape latency of the rats to find a visible platform in the water maze, no difference was observed among the different groups. Furthermore, rats injected with comparable concentration of *E.coli* filtrate did not show learning and memory deficit, suggesting that the effect of *H. pylori* filtrate on cognition is specific. Several clinic investigations have reported that the severity of *H. pylori* infection is correlated with cognitive performance of the normal adults and MCI (mild cognitive impairment, a prodromal phase of AD) patients (Kountouras et al., 2007b; Beydoun et al., 2013), and eradication of *H. pylori* is associated with decreased progression of dementia (Kountouras et al., 2009b; Chang et al., 2013) in AD patients. Our study provided the first laboratory evidence that *H. pylori* could induce AD-like spatial learning and memory impairment.

To disclose the underlying mechanism for the behavior deficit, we detected the neuronal numbers in hippocampus and cortex, two brain regions responsible for spatial learning and memory. No change was observed in the rats injected with *H. pylori* filtrate, indicating that *H. pylori* did not induce neuronal death. Thus, the learning and memory impairment in *H. pylori* filtrate-injected rats may be resulted from disturbance of neuronal function. Aβ level is increased in AD brains and induces cognitive deficit in AD animal models (Billings et al., 2005; Liu et al., 2013). Considering the correlation of *H. pylori* infection and AD, we suspect that *H. pylori* may increase the production of Aβ, and then promote the cognitive dysfunction. To test this hypothesis, we detected Aβ_40_ and Aβ_42_ levels both in the hippocampus and cortex of the rats. A significant elevation of Aβ_42_ was observed in hippocampus and cortex of the rats injected with *H. pylori* filtrate. Compared with Aβ_40_, Aβ_42_ is more easily to form aggregates (Jarrett et al., 1993), and more toxic to neurons (Zhang et al., 2002). Intracerebroventricular injection of Aβ_42_ induces memory impairment in water maze and passive avoidance tests in mice (Jhoo et al., 2004). In AD transgenic mice, Aβ_42_ increases to a higher level than Aβ_40_ and correlates with the cognitive deficits (Hsiao et al., 1996; Billings et al., 2005). More importantly, intracellular Aβ_42_ but not Aβ_40_ accumulation in AD-vulnerable brain regions is an early event preceded both NFT and plaque deposition (Iwatsubo et al., 1994; Gouras et al., 2000). Thus, peripheral *H. pylori* infection may cause learning and memory deficit through enhancing Aβ_42_ production in rat brains. Consistent with the behavior deficits, retarded dendritic spine maturation, and decreased membrane expression of learning/memory related synaptic receptors and scaffolding proteins were observed in *H. pylori*-injected rats. Aβ peptides may disrupt hippocampal synaptic plasticity via altered NMDA or AMPA receptor-PSD-MAGUK interactions (Proctor et al., 2011), these data further confirmed our speculation that *H. pylori* filtrate causes cognitive damage through Aβ_42_.

Aβ is formed by sequential cleavage of APP by β and γ-secretase (Selkoe, 1998). To explore the mechanisms underlying the *H. pylori*-induced Aβ elevation, we detected the expression levels of key functional proteins in β and γ-secretase such as BACE-1, PS-1, and PS-2. A significant increase of PS-2 in *H. pylori* filtrate-injected rat hippocampus and cortex was observed, indicating that *H. pylori* filtrate may promote Aβ_42_ production by enhancing the activity of γ-secretase. Enhanced cleavage of APP by γ-secretase is the key event inducing Aβ overproduction; inhibiting the γ-secretase is considered to be the leading amyloid-based approach to preventing AD (Selkoe, 1998). In familial early-onset AD, more than 50 missense mutations in PS-1 and PS-2 have been found, and they selectively increase the production of Aβ_42_ (Citron et al., 1997; Xia et al., 1997; Qi et al., 2003). In our experiment, *H. pylori* filtrate injection resulted in increased Aβ_42_ production and PS-2 expression, strongly indicating that *H. pylori* could promote amyloidosis partially through targeting PS-2. To further confirm this hypothesis, we incubated N2a/APP cells with *H. pylori* filtrate directly, and observed the same results: *H. pylori* filtrate incubation not only increased intracellular Aβ_42_ level but also promoted Aβ_42_ release, with a simultaneous up-regulation of PS-2 in cells. Thus, soluble exotoxins, or surface proteins released from the *H. pylori* bacteria may directly promote Aβ_42_ production and release by enhancing the activity of *γ*-secretase. The precise mechanisms for how the soluble surface fractions of *H. pylori* get into the brain and influence the neurons need further investigation. Furthermore, which component in the *H. pylori* filtrate contributes to the above described effects also needs further exploration. Another possibility is that *H. pylori* filtrate may induce a peripheral inflammatory response such as production of cytokines; the latter may further initiate the pathological changes in the brain. But in our recent study (in revision), two cytokines (TNF-α and IL-8) which were identified to be increased in AD plasma (Roubaud-Baudron et al., 2012) showed no change in *H. pylori* filtrate-injected rats. Thus, other inflammatory mechanisms should be explored.

In a summary, we have found in the present study that injection of *H. pylori* filtrate increases Aβ_42_ production with elevated expression of PS-2. *H. pylori* filtrate leads to spatial learning and memory deficits in the rats, and impairs the synaptic maturation. Our data have provided molecular evidence to disclose the intrinsic link between *H. pylori* infection and AD-like Aβ overproduction and memory impairments.

## Author contributions

Conception of the research: Ji Zeng and Rong Liu. Performing experiments: Xiu-Lian Wang, Ji Zeng, Jin Feng, Yi-Tao Tian, Yu-Jian Liu, Mei Qiu, Xiong Yan, Yang Yang, and Qun Wang. Analyses and interpretation of results: Yan Xiong, Zhi-Hua Zhang, Jian-Zhi Wang, and Rong Liu. Drafting of the manuscript: Xiu-Lian Wang. Critical revision of the manuscript: Rong Liu, Ji Zeng, and Jian-Zhi Wang.

### Conflict of interest statement

The authors declare that the research was conducted in the absence of any commercial or financial relationships that could be construed as a potential conflict of interest.
